# Development of and User Feedback on a Board and Online Game to Educate on Antimicrobial Resistance and Stewardship

**DOI:** 10.3390/antibiotics11050611

**Published:** 2022-05-01

**Authors:** Diane Ashiru-Oredope, Maxencia Nabiryo, Andy Yeoman, Melvin Bell, Sarah Cavanagh, Nikki D’Arcy, William Townsend, Dalius Demenciukas, Sara Yadav, Frances Garraghan, Vanessa Carter, Victoria Rutter, Richard Skone-James

**Affiliations:** 1 Commonwealth Pharmacists Association, London E1W 1AW, UK; maxencia.nabiryo@commonwealthpharmacy.org (M.N.); sarah.cavanagh6@nhs.net (S.C.); nikki.darcy@commonwealthpharmacy.org (N.D.); sara.yadav@commonwealthpharmacy.org (S.Y.); frances.garraghan@commonwealthpharmacy.org (F.G.); vanessa.carter@commonwealthpharmacy.org (V.C.); victoria.rutter@commonwealthpharmacy.org (V.R.); 2 Focus Games Ltd., Glasgow G40 1DA, UK; Andy@focusgames.com (A.Y.); melvin@focusgames.com (M.B.); dalius@focusgames.com (D.D.); 3 Health and Education Trust, London NW1 4LE, UK; william.townsend@thet.org (W.T.); Richard.Skone-James@thet.org (R.S.-J.)

**Keywords:** antimicrobial resistance (AMR), antimicrobial stewardship (AMS), AMS Game, board game, online game, Commonwealth Partnerships for Antimicrobial Stewardship, CwPAMS, gaming, game-based learning, gamification

## Abstract

Antimicrobial resistance (AMR), particularly antibiotic resistance, is one of the most challenging global health threats of our time. Tackling AMR requires a multidisciplinary approach. Whether a clinical team member is a cleaner, nurse, doctor, pharmacist, or other type of health worker, their contribution towards keeping patients safe from infection is crucial to saving lives. Existing literature portrays that games can be a good way to engage communities in joint learning. This manuscript describes an educational antimicrobial stewardship (AMS) game that was co-created by a multidisciplinary team of health professionals spanning across high- and low- to middle-income countries. The online AMS game was promoted and over 100 players across 23 countries registered to participate on 2 occasions. The players were asked to share feedback on the game through a short online form. Their experiences revealed that the game is relevant for creation of awareness and understanding on antimicrobial stewardship in both high- and low-to-middle income settings worldwide.

## 1. Introduction

Antimicrobial resistance (AMR), particularly antibiotic resistance, is one of the most challenging global health threats of our time [1]. Even under the shadow of COVID-19, AMR posed a substantial threat to patients who developed secondary bacterial infections [2]. AMR causes challenges to the treatment of infections and infectious diseases, including HIV/AIDS, typhoid, cholera, tuberculosis, gonorrhoea, hospital-associated infections, and malaria, which disproportionately affect low- and middle-income countries (LMICs) [1]. AMR is also particularly prevalent and problematic in LMICs where health systems and medical resources, including access to water, sanitation, and hygiene (WASH), are limited, as well as where socioeconomic drivers, such as extreme poverty, increase the risk of communicable diseases exponentially [3]. The Fleming Fund was created in response to this need, and has funded many programmes of work, including the Commonwealth Partnerships for Antimicrobial Stewardship Programme (CwPAMS). CwPAMS addresses AMR through antimicrobial stewardship interventions in eight African Fleming Fund priority countries, using a health partnership approach.

Tackling AMR requires a multidisciplinary approach. Whether a clinical or healthcare team member is a nurse, doctor, pharmacist, cleaner, or other type of health worker, their contribution towards keeping patients safe from infection and the spread of resistant organisms is crucial to saving lives.

Existing literature portrays that games can be a good way to engage communities in joint learning [4,5]. Evidence shows that games have been used to promote health and wellbeing in regard to both infectious and non-infectious diseases [6]. As a health education tool, games have proven to be an enjoyable method that enhances learning through stimulating players’ interests and motivation [6,7]. While existing studies largely point to games as an impactful educational tool for children and students, some studies have demonstrated that games can be used as a capacity-building intervention for health professionals [8]. Games can be used for improving health professionals’ knowledge and skills, changing their attitudes and performance, and improving how they care for their patients [8]. Despite the strong indication that games can improve knowledge, several studies including those conducted among health professionals do not show sufficient evidence that games can improve performance or change behaviour [7,9,10,11]. To this end, there is a need for more research with a focus on outcomes that go beyond knowledge assessment, to include outcomes such as skills, behaviour, and patient outcomes [10].

Few existing studies highlight that AMS games can be an innovative way of spreading awareness on AMR. A study conducted in Saudi Arabia among students concluded that gamification using an AMS board game can significantly improve AMR knowledge, with better retention than a conventional lecture [12]. In another study conducted in the UK among children aged 7–15 years, it was reported that antibiotic games improved knowledge on the use of antibiotics for bacterial versus viral infections and ensured that the course of antibiotics was completed [13]. The antimicrobial stewardship (AMS) game described in this manuscript was developed to encourage players (healthcare teams including students, doctors, laboratory staff, pharmacists, and nurses) to discuss AMS and learn what they can do personally, and collectively, to improve stewardship in their organisation and their community. The intention was to create a game for groups to play, and in order to make the game more widely accessible, a physical printed tabletop board game was developed with an online version for groups working remotely. Further, it is intended that the game would encourage the player to understand the scale of the AMR problem and that everyone who uses, dispenses or prescribes antimicrobials is part of the solution to reduce the impact of AMR. Effective training and capacity building are vital to the success of stewardship programmes, particularly when staff are new to the concept. The AMS game is intended to make stewardship training engaging and inclusive, generating fun and enthusiasm with a serious purpose and clear outcomes.

Focus Games Ltd. was the game development partner for this project. Focus Games Ltd. has been a leading developer of ‘serious’ educational games/game-based learning and simulations since 2004. They have developed over 100 different games for staff, patients, and the public that address a wide range of clinical, health, and wellbeing issues.

Independent evaluations of these games demonstrate that they can improve knowledge and encourage beneficial changes in thinking and behaviour [14].

Subject matter expertise, technical competency, and decision-making are diluted when individuals cannot communicate effectively with the people around them. These interpersonal skills can be learned and developed in the same way that subject matter expertise is developed. However, because these interpersonal ‘soft’ skills are largely intangible, we need to find appropriate ways of teaching.

Although there is not a definitive theoretical framework for the development of serious games, key elements of good practice have previously been summarised [15]. In development of the AMS game several elements of published good practice [15], were considered for incorporation:

- Competition and goals, with players competing against other players both for the physical and online games.
- Clear rules that define how the game is played.
- Choice through the use of multiple-choice questions.
- Challenges—players are provided with problems to solve in this game. We use the case studies as a way to provide additional challenges.
- Coaching, debriefing, and feedback: to reinforce learning.
- Performance assessment, so players know how they did.
- Mechanics: the elements of the game that control gameplay.

The development of the AMS game involved a wide range of stakeholders from across the globe, who represented the target audience for which the game was being developed. To test the usability of the developed AMS game, we used online platforms to invite interested individuals including healthcare teams to play the AMS game and asked them to share their experiences through an online feedback survey form. 

## 2. Materials and Methods

The game was developed by the Commonwealth Partnerships for Antimicrobial Stewardship Programme (CwPAMS), led by the Commonwealth Pharmacists Association (CPA) and Tropical Health and Education Trust (THET) in partnership with Focus Games Ltd. The CPA was the overall technical lead for developing the antimicrobial stewardship concepts (questions and answers) used in the game. Focus Games Ltd. was responsible for programming the AMS concepts into a playable game. THET’s coordination expertise was leveraged in bringing together relevant partners to support the development of the game.

### 2.1. AMS Game Development

**Figure 1:**
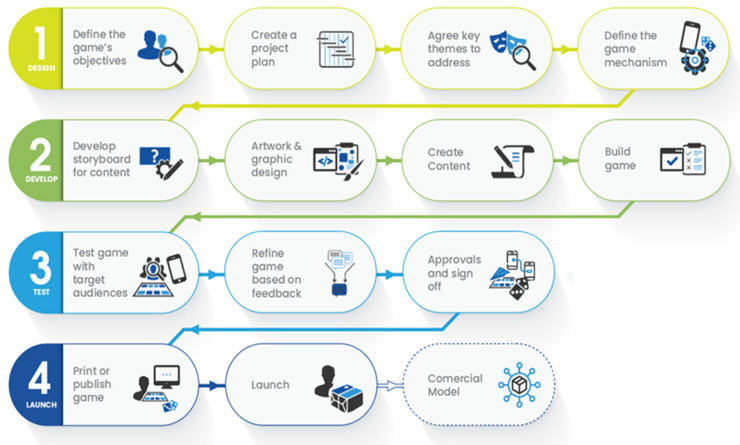
Focus Games Ltd’s Game development process/pathway [16].

The AMS game used the Focus Games Ltd. development process/pathway (Figure 1).

In order to optimise relationships and collaboration between stakeholders and programme team, Focus Games Ltd. used a range of project management tools to ensure that resources and priorities were managed efficiently and that they were overseen by a programme manager.

At the beginning of the project, a project plan and milestones were agreed upon as well as a specification document which included deliverables pertinent to the project. These deliverables formed the sign-off and approval for the completion of the project.

Focus Games used agile project management processes and tools (Jira). All code development was managed in Bitbucket (GIT repository), ensuring a strong development, test, and live approach. All staff are suitably qualified in their respective disciplines.

The development process for the AMS game included four key phases previously developed by Focus Games Ltd. (Figure 1).

Phase 1—Define Objectives: Requirements for the game outcomes were derived from interviews with and feedback from individual stakeholders, ranging from national to frontline health professionals including pharmacists, doctors, and nurses from eight African countries and the UK. They acted as subject matter experts as well as representatives for the target audience of the game through the development and user testing phases, which included syntax and grammar that was considered acceptable across multiple countries. This identified the learning objectives for the narrative structure of the factual content and how the game design and mechanism will facilitate engagement and learning.

Phase 2—Develop Storyboards: Specific learning objectives and game dynamics were identified based on best practice derived from experience of previous games developed by Focus Games Ltd. This involved the creation of graphic design mock-ups of the game and a written storyboard outline of the factual content. These were discussed and agreed upon by the project team, which included subject matter experts as well as stakeholders across nine countries (Ghana, Kenya, Malawi, Nigeria, Sierra Leone, Tanzania, Uganda, United Kingdom, and Zambia) that were all part of the countries of focus for the CwPAMS programme.

Phase 3—Prototype and Testing: A rapid prototyping approach was used, incorporating data from the pilot scenario and interviews with subject matter experts (SMEs). The game mechanism and design were finalised. The written content was drafted and loaded into the game mechanism. The game was subsequently tested in workshops with SMEs whose feedback informed design iterations.

Phase 4—Refine, Rework, and Launch: production of the final design and implementation of the distribution and commercialisation strategy.

The model and process used are based on practical experience of developing 100 educational games that are being actively used by healthcare professionals around the world. Focus Games have previously evaluated third-party frameworks and models and have found many of them impractical for the purpose of developing game-based learning for the healthcare setting.

### 2.2. Target Audience for the AMS Game

The AMS game is intended to support education on antimicrobial stewardship among current and future healthcare team members, including doctors, nurses, pharmacy teams, laboratory staff, and students.

### 2.3. Stakeholder Engagement

The game was co-created between partners in the UK and eight African countries that were part of the CwPAMS programme to ensure that the game is relevant, effective, and was designed to be used in either a high-income or low- to middle-income setting. The four broad areas addressed in the game are introduction to antimicrobial resistance and stewardship, appropriate use of antimicrobial agents, infection prevention and control, and stewardship and surveillance.

Partners and their network of health professionals were asked to share insights on the game. Twenty-seven stakeholders ranging from national to frontline health professionals, including pharmacists, doctors, and nurses across nine countries (Ghana, Kenya, Malawi, Nigeria, Sierra Leone, Tanzania, Uganda, United Kingdom, and Zambia), were consulted on the type of the game—snakes and ladders, the question cards for the AMS game, and case studies to include in the AMS game. Stakeholders were asked to share broad comments about content and accuracy of the answers to the questions in the game within their context and also asked to suggest any additional questions. To guide the feedback process, a structured feedback form (available as Supplementary File S1) was developed in Microsoft Word and shared with the stakeholders via email. The feedback form covered the following sections: demographics of the respondent (name, title, country, profession, job title, and email address), relevancy of the questions to the country, responsiveness of the questions to the key aspects of AMS, and also provided space for them to suggest other potential topics for inclusion in the game.

**Figure 2:**
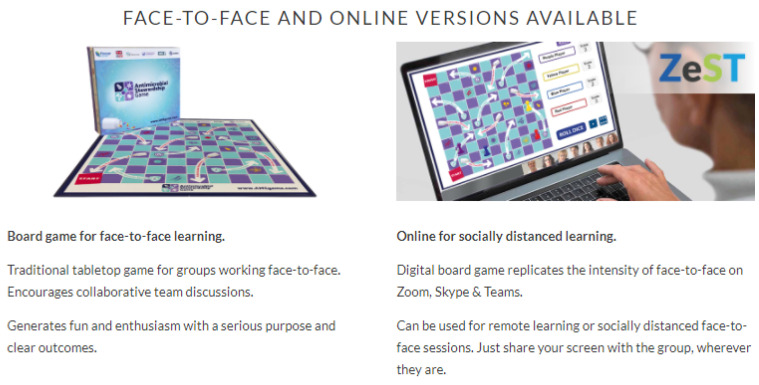
Board and online AMS games [17].

Demonstration of the board and online games (Figure 2) are available via https://commonwealthpharmacy.org/press-release-launch-of-the-antimicrobial-stewardship-ams-game/ accessed on 27 April 2022.

A guide was developed for the facilitators to provide direction on how to host the game (available in Supplementary File S2). Sample questions and answers are available in Supplementary File S3. A prize was planned for the participants of the game. The participants who completed the evaluation form of the game were entered into a draw to win access to the online AMS game for a period of one year

### 2.4. Recruitment of Participants and Playing the Game

The AMS game was first launched and played in August 2021, and then played again during the World Antimicrobial Awareness Week in November 2021 as part of a global tournament (Supplementary File S4). On both occasions, the game was promoted globally through online channels including email, websites, and social media to encourage people across the globe to register as players or facilitators. The facilitators had a technical background in antimicrobial resistance and stewardship and the project team provided a short briefing session on how to steer the session between the playing teams.

The game was hosted on Zoom, where participants received a Zoom link upon registration and used it to join the game session. Players were allocated to different breakout rooms with facilitators. In each breakout room, players were divided into two teams. Teams took turns to answer and discuss a series of questions and case studies about AMR and AMS that were being shown after the facilitators rolled the dice. The game lasted for 45 min. The facilitators were responsible for organizing players into teams, and moderating the game by displaying questions, encouraging players to discuss the questions and agree on the response, and displaying the correct answer after the players’ responses. The facilitator guide is available as Supplementary File S2.

### 2.5. Feedback from Players of the Game

We collected feedback from individuals who played the AMS game on 24 November 2021. The feedback was collected using an online questionnaire with quantitative and open/free response text-based options hosted on Survey Monkey. This was a short questionnaire with 13 questions that: collected the players’ demographic information, examined their experience in antimicrobial stewardship, assessed their knowledge gain and confidence in antimicrobial stewardship after playing the game, and assessed their perceptions on the game in terms of enjoyment and whether they would share lessons from the game and also recommend it to others.

### 2.6. Data Management

Data were collected anonymously, although survey respondents could voluntarily provide their name and email address should they wish to be contacted afterwards, e.g., for information about game prize winners and future relevant AMR events. All data were anonymised prior to data analysis. The data were held securely by the project team and in line with the General Data Protection Regulation 2016/679 [18].

### 2.7. Data Analysis

Descriptive statistics on the frequency distributions and percentages were used to analyse the responses. Data were analysed using Stata 14.

## 3. Results

### 3.1. Developing the Game: Feedback on Relevance

#### 3.1.1. Demographics of Respondents

Twenty-six stakeholders ranging from national to frontline health professionals, including pharmacists [18], doctors [4], nurses [3], and an epidemiologist [1], across nine countries (Kenya 3, Malawi 3, Nigeria 3, Sierra Leone 3, Tanzania 2, United Kingdom 4, Uganda 3, and Zambia 6), provided responses on the relevance of the game (Table 1).

#### 3.1.2. Feedback on Relevance of Questions of the Game

When asked whether the questions were broadly relevant to their countries, all 25 respondents who answered this question said “yes”. However, 40% of the 25 respondents mentioned that some questions were not at all relevant or were inaccurate to their countries (Table 1). To this end, respondents shared some of the following suggestions, including questions that could be added to the game:

“*What is antibiotic resistance? The response needs to be modified to include inappropriate antibiotic use as a driver of antibiotic resistance not optimal use of the same. The response should also include that antibiotics are not effective against parasites*.”

“*Perhaps consider adding some more questions on the role of IPC and the MTC in AMS*.”

“*I would suggest to add the following topics on hospital-associated infections. Diagnostics on how to identify the presence of a microbe in a patient specimen*.”

On the other hand, the questions and case studies were found to address the key aspects of AMS, as indicated by all 24 respondents that answered the question.

### 3.2. Players’ Feedback on the AMS Game

#### 3.2.1. Demographics of Respondents

In total, 328 individuals from 23 countries registered to join the online game sessions at the launch of the game in August 2021, and during World Antimicrobial Awareness Week in November 2021. More than 120 attended the two live sessions and 74 participants responded to the AMS game evaluation form. The respondents represented 13 countries across 4 regions: Africa [7], Europe [2], South-East Asia [2], and the Western Pacific region [1] (Table 2). More than half (62.2%) of the respondents were pharmacists. The majority of respondents had experience in AMS, with 20.3% recorded as AMS specialists. However, 10.8% of the respondents were new to AMS.

#### 3.2.2. Player Enjoyment of the AMS Game

When asked whether they enjoyed playing the AMS game, almost all (91.9%) of the respondents agreed that they enjoyed it (Table 3). The reasons for enjoyment were mainly attributed to: the simplicity of the design of the game, making it easy to play, and the game was viewed as a novel, unique, interactive, and fun approach for empowering participants to tackle AMR. Comments included:

“*The game was highly entertaining and enriching. You get to learn more of the AMS or rather, fine-tune your knowledge via the game. Will really love to partake again. Kindly keep me posted. Thanks*.”

“*The game was very interesting, straight forward and all-round included the One Health aspect which I am really interested in that I think is the way to go if mitigation of antimicrobial resistance is to be achieved*.”

“*The game is a good way of bringing the awareness to health workers. It should be rolled out across and include students in medicine, nursing, pharmacy, and laboratory*.”

“*What I loved most was that the answers provided by the game were simple, understandable and straightforward to the point and also there was more valuable information attached to the answer thus giving more understanding and meaning*.”

On the other hand, time was a common reason stated as a limitation to enjoyment of the game. The reason for this was attributed towards the short time allocated for the demonstration and not the game itself, whereby players would usually enjoy the game at their own pace. In this perspective, respondents felt that if given more time, the game would be a valuable tool for learning.

“*The demonstration was a bit rushed so difficult to get a real feel for the game. I can see it could be a useful tool to facilitate discussion around AMS with more junior/inexperienced staff. The game needs to be seen in this light because I feel if the focus becomes on playing the game, answering questions as quickly as possible, etc., then it’s true value will be lost*.”

“*A good fun, learned few things for a very short time*.”

“*Cross talking among participants making it difficult to respond…time constraints…facilitators should not talk much*.”

#### 3.2.3. Knowledge Gain after Playing the Game

The majority (75.7%) of respondents agreed that they got to know more about AMS after playing the game (Table 4). In this regard, respondents from the African Region agreed more than respondents from the European Region by a difference of 12.1% (Table 5). Qualitatively, respondents mentioned to mainly have gained knowledge on the relevance of One Health in AMS and others perceived the game as an opportunity to refresh the principles and strengthen their knowledge on AMS.

“*I understood that not only human health must be emphasised so as to end antimicrobial resistance but also animal and environmental health. Interacting with more informed players was more informing*.”

“*The game was a quick mind check for the principles on AMR*.”

Further, when asked to highlight the most important things they learned from the game, respondents commonly stated aspects related to improved understanding of One Health and handwashing as key aspects in AMS. Additionally, respondents acknowledged the gain of understanding of key AMS terminologies, such as “biosecurity” and “watchful waiting”. Overall, there were expressions of increase in knowledge on the causes and preventive measures of AMR. It was also realised that there is a need to create more awareness on AMR and AMS and it was commonly stated that games can be a fun and interactive approach for empowering health professionals and other team members to improve AMS.

“*Actually, it’s funny to say but today I learnt that there is a difference between antibiotics and antimicrobials. At first, I thought there was no difference. And I have also learnt about antimicrobial and antibiotic resistance*.”

“*I learned that simple practices such as handwashing play a big role in fighting AMR*.”

“*Learning is fun when made simple in such innovative ways. I also think it can stick to the brain and allows easy replication in actual practice settings*.”

Relatedly, more than three quarters (79.5%) of respondents expressed having more confidence about AMS after playing the game. Respondents associated gaining confidence with acquiring knowledge from the game, thus making them better positioned to create awareness on AMS, as explained by the participants’ comments below:

“*I can now educate my colleagues, the nation and the entire world about the goodness of antimicrobial stewardship*.”

“*I think the game is great, since am just an undergraduate student but I have been able to reason out different things with people already in the profession. This has boosted my confidence*.”

#### 3.2.4. Sharing Lessons Gained from Playing the Game

The majority (93.3%) (Table 6) of participants responded in agreement about sharing lessons from the game with colleagues. Some voiced reasons as to why they would share lessons and others mentioned platforms they would use for sharing, such as those highlighted below:

“*I have to share what I have learnt with others so as to facilitate continuation of learning and flow of information*.”

“*I will share during our grants meetings and AMS meeting*.”

“*I will promote for use in WAAW (World Antimicrobial Awareness Week)*.”

“*It was such an engaging experience and will be helpful for bonding in our hospital teams*.”

Concerning sharing lessons from the game with patients, more than half (72.6%) (Table 6) of the respondents expressed willingness to do so for some of the following reasons:

“*Completing one’s dosage as prescribed by a trained health personnel helps minimise antimicrobial resistance*.”

“*The patients can use the game to learn more about AMS*.”

Further, almost all (97.3%) of the respondents mentioned that they would recommend the game to their colleagues (Table 6).

## 4. Discussion

This paper has documented the process of developing the AMS game, and reflections by players. Overall, the healthcare teams who played the game found it enjoyable and reported that it can impart knowledge and confidence in AMS and facilitate shared learning with colleagues and patients.

### 4.1. AMS Game Development

The AMS game was developed to attract a multidisciplinary team of players to discuss AMS and learn what they can do personally, and collectively, to improve stewardship in their organisation and community. A multi-country and multidisciplinary approach was used in the development of the game. This supported incorporation of the contextual educational needs of both high- and low- to middle-income countries as shared by diverse health professionals, including pharmacists, nurses, and doctors from Africa and Europe. With this, we addressed the gap reported in a scoping review on serious health education games targeting healthcare providers, patients, and public health users, where it was reported that LMICs have rarely been considered in the development of games [18]. Consequently, the AMS game described in this manuscript attracted diverse participants, players, and facilitators across 23 countries. Further, it is recommended that for effectiveness, games should be designed according to the requirements of specific groups of people [19]. The AMS game was designed for teams of healthcare staff at all levels. To cater for the needs of diverse staff members, the game was designed with different levels of complexity so the organiser can select questions to match the learning requirements of each group.

### 4.2. Players’ Feedback on the Game

The results showed that the game was enjoyable, as indicated by almost all (91.9%) participants who shared feedback. As in other studies, the enjoyment of the game was attributed to its simplicity in design, making it easy to understand and play [13,19]. The AMS game was also found to be interactive and thus a component that participants enjoyed. The component of playing the game in teams encouraged discussion and learning among players, which also made it more engaging. A systematic review that explored user engagement features in digital games documented more engaging features that can be considered in digital games: the game should have an attractive storyline, be adaptable to gender and age, high-end realistic graphics, well-defined instructions, in combination with clear feedback and a balance of educational and fun content [20]. Whilst the AMS game described in this manuscript covered all these features in its design and appearance, the gender and age components were not considered and neither did the evaluation of the game cater for these variables. It would be important to explore such in future.

It was evidenced that the game can translate knowledge and create awareness on AMS among the players. Higher knowledge gain was recorded among participants from the African countries compared to those from the European countries by a difference of 12.1%. This could imply that the game is more beneficial to the African countries where there are lower levels of awareness on AMR and AMS, as indicated in several studies [12,13,14,15,16,17,18,19,20,21,22,23]. It was clear from the study findings that the role of One Health in AMS was one of the most common key lessons gained by players, and this is likely because many of the respondents focused on human health in healthcare settings and likely had less exposure to One Health principles. To this end, the AMS game was relevant in accelerating knowledge on the One Health approach, which is recommended by the WHO as a strategy for health workers to responsibly use antimicrobials [24].

The feedback on the game did not only further expose the inadequacy in knowledge on key terminologies in AMS among health professionals, but instantly created awareness and knowledge on terms such as ‘antimicrobial stewardship’, ‘biosecurity’, and ‘watchful waiting’. Therefore, the AMS game could be a solution to challenges discovered in studies such as that of Higuita-Gutiérrez et al. [25], where 81.8% of 532 medical students had never heard of the term ‘antimicrobial stewardship’. Generally, this AMS game may have the potential to increase awareness among health students and professionals across the globe. It can be considered as part of a wider AMR education programme within universities and health institutions. The online version provides an opportunity for use across multiple institutions.

Following the AMS game tournaments, a substantial portion (75.9%) of players who provided feedback indicated that they gained confidence in AMS and thus would participate in creation of awareness on AMS thereafter. This was further demonstrated when over 90% of the study respondents indicated that they would share lessons from the game with colleagues and patients.

### 4.3. Strengths and Limitations

Our AMS game was developed with consideration of perspectives from a multidisciplinary team of professionals across the UK and African countries. Hence, this game does not only promote One Health in antimicrobial stewardship but is also among the few games that cater for the antimicrobial stewardship awareness needs of both high- and low- to middle-income countries. Whilst the study results indicated that our AMS game is very likely to improve awareness and understanding of AMS, as a limitation, it should be noted that this was a small study designed to collect initial feedback on the new board and online AMS game. Further, only subjective measurements were used to collect feedback on the educational potential of this AMS game, and the small number of players (respondents) makes statistical analysis infeasible. As such, the results of the current study need to be strengthened by studies employing stronger methodologies. We recommend that future studies consider a randomized controlled trial utilizing objective measurement to evaluate the AMS game among a higher number of respondents sampled from a group of individuals who played the game. It would be important that future studies consider: a greater representation of other types of health professionals as the current study had much higher representation ofpharmacists, evaluate the impact of the players’ discussions in the breakout rooms (which was not evaluated in this study), and study the effectiveness of the AMS game in comparison to other AMS education interventions.

Despite these limitations, this study generated more evidence and demonstrated that a board and online AMS game is likely to improve knowledge of antimicrobial stewardship. The AMS board game continues to be promoted, made accessible, and used as an education tool among health professionals [26].

## 5. Conclusions

We provide a documentation of the process of developing a board and online game on antimicrobial resistance and stewardship; and its potential to educate diverse health care teams in high or low-to-middle- income countries. The game was co-created with a diverse group of stakeholders including national and frontline health professionals from high- and low-income countries. The feedback from the initial players (health professionals) of the game highlighted that the game is enjoyable. Also, that it provides an innovative and engaging opportunity for the players to discuss topics in AMR and AMS; whilst improving and strengthening their knowledge of key topics. Further studies will be useful in evaluating the impact of the AMS game as an educational tool for antimicrobial resistance and stewardship.

## Figures and Tables

**Table 1:** Respondents’ feedback on relevance of questions.

	Number of Respondents	Percentage (%)
**Questions broadly relevant to your country**	***n* = 25**	
Yes	25	100
No	0	0
**Some questions not at all relevant or inaccurate for your country**	***n* = 25**	
Yes	10	40
No	15	60
**Questions and case studies address key aspects of AMS**	***n* = 24**	
Yes	24	100
No	0	0

**Table 2:** Demographics of respondents (players’ feedback).

**Country**	**Number of Respondents (*n* = 74)**	**Percentage (%)**
**European Region**	39	52.8
United Kingdom	38	51.4
Hungary	1	1.4
**South-East Asia Region**	2	2.7
India	1	1.4
Sri Lanka	1	1.4
**African region**	32	43.2
Uganda	15	20.3
Kenya	7	9.5
Ghana	2	2.7
Nigeria	3	4.1
Sierra Leone	3	4.1
Eswatini	1	1.4
Malawi	1	1.4
**Western Pacific Region**	1	1.4
Fiji	1	1.4
	**Number of respondents (*n* = 74)**	**Percentage (%)**
Role/Profession		
Pharmacist	46	62.2
Student	7	9.5
Doctor	6	8.1
Other (Academic, Environmental Health)	5	6.8
Support staff	4	5.4
Other clinical staff	3	4.05
Laboratory staff	2	2.7
Nurse	1	1.4
**Level of Experience in AMS**		
Some knowledge of AMS	30	40.5
Experienced in AMS	21	28.4
Specialist in AMS	15	20.3
New to AMS	8	10.8

**Table 3:** Enjoyment playing the AMS game.

Enjoyed Playing the AMS Game	Number of Respondents (*n* = 74)	Percentage (%)
Strongly disagree	2	2.7
Disagree	0	0
Neither disagree nor agree	4	5.4
Agree	26	35.1
Strongly agree	42	56.8

**Table 4:** Knowledge and confidence gain after playing the game.

	Number of Respondents	Percentage (%)
**Know more about AMS after playing the game**	*n* = 74	
Strongly disagree	6	8.1
Disagree	3	4.1
Neither disagree nor agree	9	12.2
Agree	35	47.3
Strongly agree	21	28.4
**More confident about AMS**	*n* = 73	
Strongly disagree	4	5.5
Disagree	3	4.1
Neither disagree nor agree	8	11
Agree	41	56.2
Strongly agree	17	23.3

**Table 5:** Knowledge gain according to geographic region.

Knowledge Gain	Region			
	African	Europe	South-East Asia	Western Pacific
Strongly disagree	3 (9.3%)	3 (7.7%)	0 (0%)	0 (0%)
Disagree	1 (3.1%)	2 (5.1%)	0 (0%)	0 (0%)
Neither disagree nor agree	2 (6.3%)	7 (17.9%)	0 (0%)	0 (0%)
Agree	14 (43.8%)	19 (48.7%)	1 (50%)	1 (100%)
Strongly agree	12 (37.5%)	8 (20.5%)	1 (50%)	0 (0%)
**Total**	32 (100%)	39 (100%)	2 (100%)	1 (100%)

**Table 6:** Sharing lessons gained after playing the game.

	Number of Respondents	Percentage (%)
**Sharing lessons with colleagues**	*n* = 74	
Strongly disagree	2	2.7
Disagree	0	0
Neither disagree nor agree	3	4.1
Agree	38	51.4
Strongly agree	31	41.9
**Sharing lessons with patients**	*n* = 73	
Strongly disagree	3	4.1
Disagree	0	0
Neither disagree nor agree	17	23.3
Agree	34	46.6
Strongly agree	19	26
**Recommending game to colleagues**		
No	2	2.7
Yes	71	97.3

## Data Availability

Data are contained within the article or Supplementary Material.

## Supplementary Materials

The following supporting information can be downloaded at: https://www.mdpi.com/article/10.3390/antibiotics11050611/s1. Supplementary File S1: Questions and comments review: Antimicrobial Stewardship Game. Supplementary File S2: A facilitator’s guide was developed to guide the facilitator’s on how to run the game. Supplementary File S3: Sample questions and answers from the game cards. Supplementary File S4: Poster for the global tournament.
