# Supplementary material for: Development of and User Feedback on a Board and Online Game to Educate on Antimicrobial Resistance and Stewardship
Source: Antibiotics (Basel). 2022 May 1;11(5):611. doi: 10.3390/antibiotics11050611 (PMC9138161; doi:10.3390/antibiotics11050611)
Supplement: Supplementary file 1 [file antibiotics-11-00611-s001.zip › Supplementary S2_AMS Game guide slides .pdf]

Antimicrobial Stewardship Game

* CwPAMS is funded by the Department of Health and Social Care using UK aid funding and is managed by the Fleming Fund. The Fleming Fund is a £265 million UK aid investment to tackle antimicrobial resistance by supporting low - and middle - income countries to generate, use and share data on AMR. The Fleming Fund programme is managed by the UK Department of Health and Social Care. CwPAMS programme is managed by Commonwealth Pharmacists Association and Tropical Health Education Trust, (THET).

Antimicrobial Stewardship Game

Play

On the day…

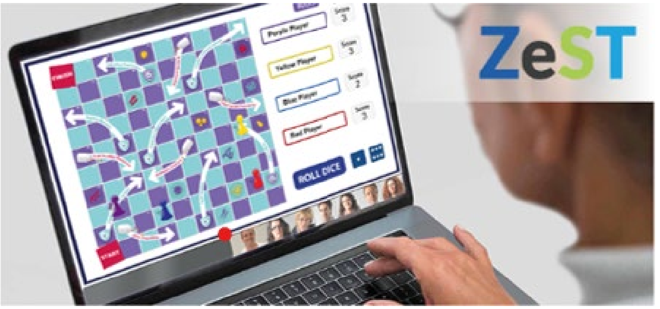

- Join the ZOOM meeting at 12:30GMT
- You will be allocated a breakout room
- Each room has a host and an assistant who will run the game
- We will play in teams
- Teams will be allocated in the breakout room
- Play will last **30- 40 minutes**
- **This is the link to the game we will use on the day (you can also use this to practice in advance)**
- **https://games.focusgames.co.uk/test/zest_ams_thursday/**

Getting started

- You as the facilitator will run each game
- Divide players into 2 teams e.g. If your birthday is an odd number join Purple team, if even number join Yellow team.
- Starting with the Purple team ask players to introduce themselves – name, country. Make a note of the members of each team.
- If the teams are uneven in number ask some players to join the other team.
- Ensure that each player is in a team (tell them to remember the team name/colour)
- Explain the rules
- In this game we encourage players to talk with each other (rather than using the chat)
- When ready start the game.

When ready - Play the AMS game:

- Roll the dice
- Player pawn moves on the board
- A Question is shown – read this out to everyone
- Encourage discussion amongst the team
- Prompt where necessary
- When time up ask for their answer
- Click the “show answer” button
- Read out the answer to everyone
- Review the answer, If correct select the green tick, if wrong click red cross
- Points will be awarded.
- Continue to the end of the game / or time up. The team with the most points wins.

Finishing the game

- The game ends when one team reaches the FINISH square

OR

- Run out of time
- The team with the highest score is declared the winner
- Click the Feedback button and display the QR code (players can use their phone to complete the feedback survey.)
- Share the URL for the feedback survey in the chat box.

- Exit the breakout room and go back to the main room for final close
- Players who complete the feedback form will be entered to the draw to win a copy of the digital AMS game.

Antimicrobial Stewardship Game

Feedback

Feedback survey links:

http://feedback.AMSgame.com
