# Supplementary material for: Development of and User Feedback on a Board and Online Game to Educate on Antimicrobial Resistance and Stewardship
Source: Antibiotics (Basel). 2022 May 1;11(5):611. doi: 10.3390/antibiotics11050611 (PMC9138161; doi:10.3390/antibiotics11050611)
Supplement: Supplementary file 1 [file antibiotics-11-00611-s001.zip › Supplementary S3_AMS Game (Qs Selected for Global Tournament).pdf]

Q

Can you name 2 infections or diseases caused by bacteria?

# A

Accept any reasonable answers, they could include:

- Urinary Tract Infection
- Tuberculosis, (TB)
- Gonorrhoea

Q

What are antimicrobials?

# A

Antimicrobials are life-saving medicines used to prevent and treat infections in humans, animals and plants. They include:

- Antibiotics
- Antivirals
- Antifungals
- Antiparasitics

Unfortunately, microbes can become resistant to the drugs and so antimicrobials stop being effective.

Q

Can you name 2 diseases or infections caused by viruses?

# A

Accept any reasonable answers, they could include:

- Colds
- Flu
- Chickenpox
- Shingles
- COVID-19
- HIV

Q

Can you name 2 diseases or infections caused by fungi?

# A

Accept any reasonable answers, they could include:

- Thrush
- Ringworm
- Athelete’s foot
- Fungal nail infection

Q

Can you name 2 diseases or infections caused by parasites?

# A

Accept any reasonable answers, they could include:

- Malaria
- Threadworm
- Head lice
- Chagas disease
- Giardia
- Leishmaniasis

Q

What is antimicrobial resistance?

A

Antimicrobial Resistance is also known as AMR. It occurs when bacteria, viruses, fungi and parasites no longer respond to medicines.

AMR means that antimicrobial medicines become ineffective and infections become increasingly difficult or impossible to treat.

AMR increases the risk of disease spread, severe illness and death.

Q

Name at least 3 things that non-healthcare workers can do to prevent and control the spread of antibiotic resistance.

# A

Answers could include:

- Regular hand washing and prepare food hygienically
- Practise safe sex
- Avoid contact with sick people and keep vaccinations up to date
- Only use antibiotics prescribed by a certified health professional
- Never demand antibiotics if you do not need them
- Always follow your health worker’s advice when using antibiotics
- Never share or use leftover antibiotics
- Only give antibiotics to animals with veterinary supervision

Q

What does *"watchful observation"* mean when we talk about patients with infections?

# A

It is a way of making sure we treat infections in the right way. The intention is to avoid using antimicrobials when they are not needed.

For example, the recommended first-line treatment for a sore throat is *watchful waiting*. If we wait a few days, the symptoms often improve or disappear without antibiotics.

Many simple and minor infections do not need medicines, the infections go away naturally. Reassure your patients that they probably do not need antibiotics.

Q

Why it is important to not use antibiotics as first-line treatment in conditions such as earache or sore throat?

# A

Not using antibiotics in such cases may help limit antibiotic resistance.

We must use the right antibiotic for a given infection, and only prescribe them when they are needed and will be effective.

Many infections do not need medicines, the infections go away naturally.

The recommended first-line treatment for earache and sore throats is *watchful waiting*. Symptoms often improve in a few days without antibiotics.

Q

What are the signs of an allergic reaction to penicillin?

# A

The signs of an allergic reaction to penicillin are:

- Raised, itchy skin rash, (urticaria or hives)
- Coughing or wheezing
- Tightness of the throat causing breathing difficulties
- Anaphylaxis
- Angioedema

These reactions are usually immediate reactions. There can also be severe delayed onset penicillin allergic reactions.

Side effects of penicillin such as nausea, vomiting or diarrhoea do not indicate an allergic reaction.

Q

Why is vaccination useful in the fight against antimicrobial resistance?

# A

Vaccination helps prevent viral and bacterial infections.

People who are vaccinated are less likely to get infections.

If they do not get infected, prescribers will not have to see them or prescribe antimicrobials.

Q

In agriculture and farming can you explain what biosecurity is?

# A

Biosecurity refers to measures aimed at preventing the introduction or spread of harmful organisms to animals and plants; in order to minimize the risk of transmission of infectious disease.

Q

Antimicrobials can be given to farm animals.

Name at least 3 things, which the agriculture sector can do to prevent and control the spread of antimicrobial resistance.

# A

Answers could include:

- Only give antimicrobials to animals under veterinary supervision
- Do not use antimicrobials with healthy animals to promote growth
- Vaccinate animals to reduce the need for antimicrobials
- Promote good practices at all steps of production and processing
- Improve biosecurity on farms
- Prevent infections through improved hygiene and animal welfare
- Educate agriculture workers on use of antimicrobials

Q

Can you name the 3 categories of antibiotics in the World Health Organization's AWaRe list?

# A

AWaRe classifies antibiotics into three stewardship groups:

1. Access: have activity against a wide range of commonly encountered susceptible pathogens and with lower resistance potential than antibiotics in the other groups.
2. Watch: have higher resistance potential. Should be prioritised as key targets of stewardship programs and monitoring.
3. Reserve: reserved for treatment of infections due to multi-drug-resistant organisms. Considered a “last resort” for highly specific patients and settings.

# Q

Name one of the WHO's "5 Moments of Hand Hygiene.“

*Do not repeat any previous answers already given.*

# A

Accept any of the following:

1. Before touching a patient
2. Before any clean or aseptic procedure
3. After body fluid exposure risk
4. After touching a patient
5. After touching patient surroundings

# Q

*Clostridioides difficile* is transmitted from person to person by:

A. Droplet contact
B. Direct physical contact
C. Airborne transmission
D. Faecal-oral transmission

# A

D is the correct answer.

*Clostridioides difficile* is transmitted from person to person by faecal-oral transmission.

Always clean your hands with soap and running water after visiting the toilet. Alcohol hand rubs are not effective against *C. difficile* spores.

Q

Name 1 type of antibiotic, which frequently causes *Clostridioides difficile* infection and Pseudomembranous colitis.

# A

Accept any of these answers:

- Cephalosporins
- Aminopenicillins
- Fluoroquinolones
- Clindamycin

**Q**

What is the correct order to remove PPE to minimize the risks of cross-contamination?

# A

You should remove your PPE in the following order:

1. Gloves
2. Gown/ apron
3. Goggles/ face shield
4. Mask/ respirator

Q

There are a number of things you must do to help stop infections spreading.

Can you name 3 of these things?

# A

Answers can include:

- Good hand hygiene. You must keep your hands clean
- Make sure all equipment is cleaned properly
- Safe disposal of waste
- Safe management of laundry
- Correct use of Personal Protective Equipment,  (PPE)
- Stay away from work if you have an infectious illness

# Q

If a patient has a flu-like illness, diarrhoea & vomiting or a chesty cough, which of the following symptoms might indicate sepsis?

A. Slurred speech or delirium
B. Severe breathlessness
C. Mottled or discoloured skin
D. Any of the above

# A

D is the correct answer.

Slurred speech or delirium, severe breathlessness and mottled or discoloured skin are all clear indicators of sepsis.

If you spot any of these, you must act quickly.

# Q

Washing with soap and running water is the best way of cleaning your hands. But you have to wash them properly.

How long should you spend washing your hands?

A. 5 seconds
B. 10 seconds
C. 20 seconds

# A

The right answer is C.

You should spend at least 20 seconds washing your hands with soap and running water.

Try timing 20 seconds so you know how long it is. Or you can hum the “Happy Birthday” song from beginning to end twice.

Q

**ACTIVITY** - Managed by the facilitator.

Ask every player to pretend to wash their hands.

# A

The facilitator should make sure that the correct hand washing procedure is demonstrated.

Correct any bad practice by demonstrating the correct procedure.

Q

In simple terms please explain what antimicrobial stewardship means.

# A

Antimicrobial stewardship is a structured way for individuals, organisations and countries to reduce antimicrobial resistance through coordinated action.

When prescribing antimicrobials consider the following:

1. The right antibiotic
2. For the right patient
3. At the right time
4. With the right dose
5. By the right route
6. Causing least harm to the patient

Q

Name 3 patient factors you should consider when you are selecting the dose of an antibiotic?

# A

When selecting the dose of an antibiotic you should consider:

- Age
- Comorbidities
    - renal function for renally excreted drugs
    - liver function for drugs which undergo hepatic metabolism
- Weight
- Therapeutic index of the drug, some narrow therapeutic index drugs, e.g vancomycin require individualised dosing as they have a narrow therapeutic window and can cause toxicity
- Published guidelines
- Concomitant medication

Q

Sometimes patients do not take their medicine in the way prescribed.

Can you give 3 reasons why this happens?

# A

Answers could include:

- The prescribed medicine is not available
- There may be no staff to get the medicine from the pharmacy
- The patient cannot afford the medicine
- The patient does not want to take the medicine
- Does not understand dispensing pharmacist’s instructions

We must find out whether the patient is taking their prescribed medicine and what the problems might be.

Good communication between healthcare professionals is essential.

**Q**

A hospital pharmacist has been asked to provide some local antimicrobial stewardship awareness training.

List 4 topic areas that could be covered in this training.

# A

Answers could include:

- Introduction to antimicrobial resistance and stewardship
- How to access and use local prescribing guidelines
- Local surveillance and antimicrobial consumption information
- WHO AWaRe list
- Surgical prophylaxis
- Antimicrobial review and decision making
- IV to oral switch guidance
- Documenting indication and review date
- Course length

Q

When writing local antimicrobial prescribing guidelines, list 3 things which should be considered.

# A

Answers could include:

- National or international, (where national guidelines do not exist), antimicrobial prescribing or infection management guidance
- Evidence based research or guidelines
- Local surveillance, if available
- Local and national resistance rates to common microorganisms
- Local availability of antimicrobials
- Cost implications of any guideline changes
- Common infections seen in the locality
- Patient population
- Opinions of local microbiology or infectious diseases experts

Q

A nurse working on a medical ward wants to help promote antimicrobial stewardship practice.

Name 3 things the nurse could do.

# A

Answers could include:

- Prompt regular reviews of IV antimicrobials
- Remind clinicians who prescribe antimicrobials to document the indication, duration and plan for antimicrobial treatment
- When laboratory results are available ensure that clinicians are aware of them, and they are used when the patients are reviewed
- Encourage colleagues to use antimicrobial prescribing guidelines
- Asking patients if they are experiencing any adverse effects
- Administer prescribed antimicrobials promptly

Q

How can a hospital pharmacist promote antimicrobial stewardship practice in their specialist area or hospital?

Name 3 things.

# A

Answers could include:

- Promote the availability of antimicrobial guidelines
- Ensure colleagues can access the guidelines
- Ensure colleagues understand how to use the guidelines
- Question prescribing of antimicrobials outside of guidelines
- Undertake local prescribing audits or quality improvement projects to assess areas where guideline compliance is poor
- Provide local educational sessions on antimicrobial stewardship
- Provide information on local antimicrobial use

Q

How can a community pharmacist promote antimicrobial stewardship practice in their pharmacy?

Name 3 things.

# A

Answers could include:

- Promote availability of antimicrobial guidelines
- Ensure colleagues know how to access the guidelines
- Ensure colleagues understand how to use the guidelines
- Question prescribing of antimicrobials outside of the guidelines
- Refuse to sell antimicrobials to the public without a prescription
- Talk to patients about the risks of antimicrobial resistance and the actions they can take to reduce antimicrobial consumption

Q

How can community health workers promote antimicrobial stewardship practice in their community?

Name 3 things.

# A

Answers could include:

- Promote availability of antimicrobial guidelines
- Ensure colleagues know how to access the guidelines
- Ensure colleagues understand how to use the guidelines
- Question prescribing of antimicrobials outside of the guidelines
- Talk to patients and public about risks of antimicrobial resistance and actions they can take to reduce antimicrobial consumption
- Advise patients not to buy antimicrobials without a prescription
- Promote principles of infection control to colleagues and the public

Q

How can a surgeon promote antimicrobial stewardship practice in their hospital?

Name 3 things.

# A

Answers could include:

- Ensure WHO Safe Surgery Checklist is used in all surgical procedures
- Develop and use local antimicrobial surgical prophylaxis guidelines
- Use single dose prophylaxis for relevant procedures
- Promote the availability of antimicrobial guidelines
- Ensure colleagues know how to access the guidelines
- Ensure colleagues understand how to use the guidelines
- Monitor rates of surgical site infections
- Address concerns, for example if high rates of infection are reported
