# Supplementary figures and images for: Development of and User Feedback on a Board and Online Game to Educate on Antimicrobial Resistance and Stewardship
Source: Antibiotics (Basel). 2022 May 1;11(5):611. doi: 10.3390/antibiotics11050611 (PMC9138161; doi:10.3390/antibiotics11050611)

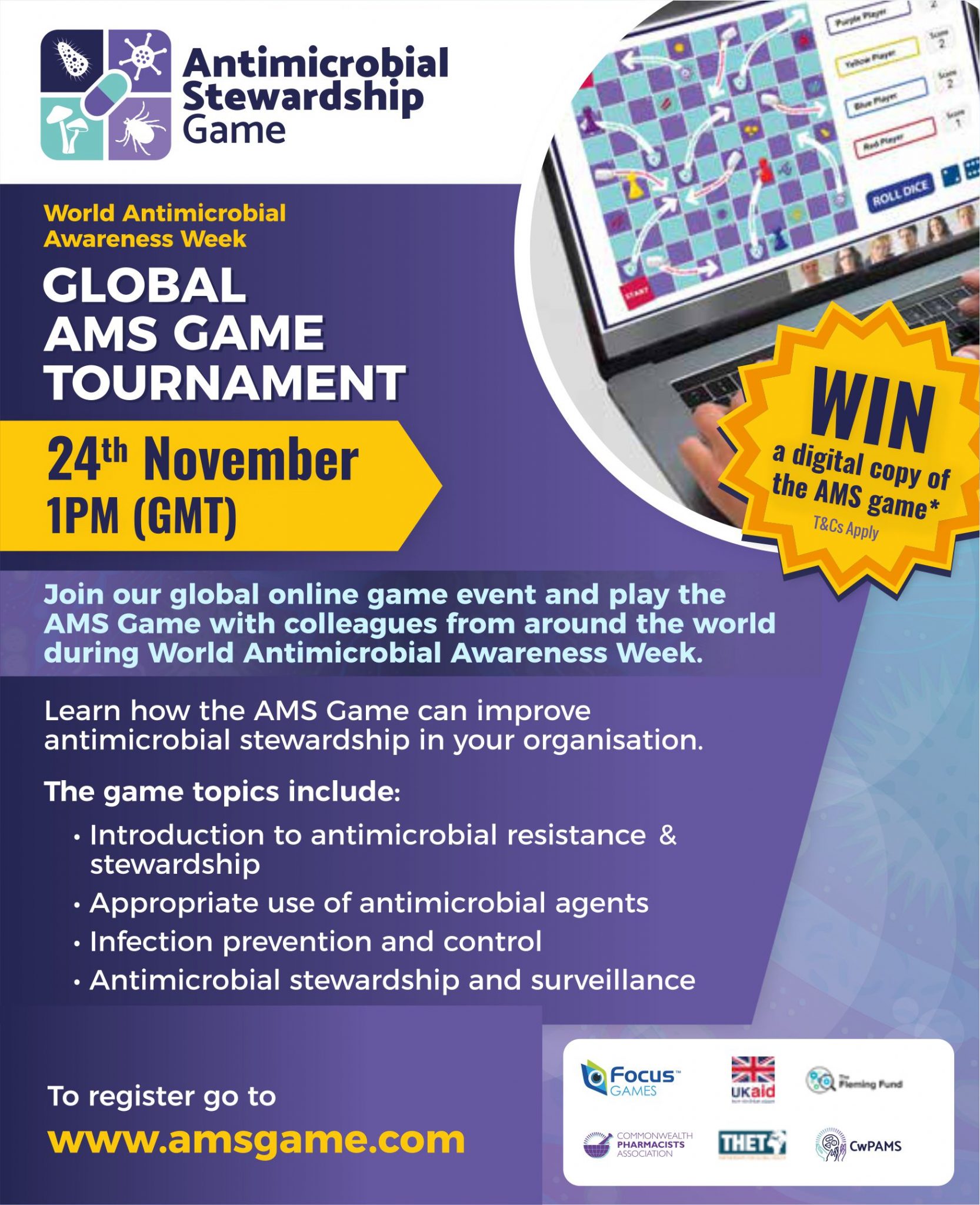

Supplement: Supplementary file 1 [file antibiotics-11-00611-s001.zip › Supplementary S4 _AMS-Game-Flyer-WAAW21-3-1666x2048.jpg]
