# Supplementary material for: Development of and User Feedback on a Board and Online Game to Educate on Antimicrobial Resistance and Stewardship
Source: Antibiotics (Basel). 2022 May 1;11(5):611. doi: 10.3390/antibiotics11050611 (PMC9138161; doi:10.3390/antibiotics11050611)
Supplement: Supplementary file 1 [file Supplementary S1_AMS Game Review Comments.pdf › Supplementary S1_AMS Game Review Comments.pdf]

CwPAMS and Focus Games are developing an educational board game to build awareness and inform healthcare professionals about Antimicrobial Stewardship. We would welcome your review of the questions to be used in the game. We are seeking broad comments about the content and accuracy of the answers within their context.

We are **not** asking you to check for spelling or grammar errors nor are we asking you to comment on the wording or layout of individual questions.

We are seeking comments that the content is factually correct, and that the topics are appropriate.

We would also welcome any suggestion on topics that could be included in the game.

We would appreciate returns by 13th May, absolute deadline 17th May 2021, email: amr@commonwealthpharmacy.org

Questions and Comments review Antimicrobial Stewardship Game

**Name, including title**:<br><br>**Country:**<br><br>**Profession:**<br><br>**Role/Job Title**:<br><br>**Email address:**
**Please answer the following questions** (free text, no word limit):
1. Are the questions broadly relevant to your country?
2. Are there any questions not at all relevant or inaccurate for your country? (please specify which)
3. Do the questions and case studies address the key aspects of Antimicrobial Stewardship?

CwPAMS and Focus Games are developing an educational board game to build awareness and inform healthcare professionals about Antimicrobial Stewardship. We would welcome your review of the questions to be used in the game. We are seeking broad comments about the content and accuracy of the answers within their context.

We are **not** asking you to check for spelling or grammar errors nor are we asking you to comment on the wording or layout of individual questions.

We are seeking comments that the content is factually correct, and that the topics are appropriate.

We would also welcome any suggestion on topics that could be included in the game.

We would appreciate returns by 13th May, absolute deadline 17th May 2021, email: amr@commonwealthpharmacy.org

4. Are there any key topics or questions that should be added?
5. Please add other suggestions for questions here:

CwPAMS and Focus Games are developing an educational board game to build awareness and inform healthcare professionals about Antimicrobial Stewardship. We would welcome your review of the questions to be used in the game. We are seeking broad comments about the content and accuracy of the answers within their context.

We are **not** asking you to check for spelling or grammar errors nor are we asking you to comment on the wording or layout of individual questions.

We are seeking comments that the content is factually correct, and that the topics are appropriate.

We would also welcome any suggestion on topics that could be included in the game.

We would appreciate returns by 13th May, absolute deadline 17th May 2021, email: amr@commonwealthpharmacy.org

There are 2 PDF files to review – one for questions (73 and one for case studies. Use the tables below to record your feedback.

Questions

Comment number	Page number<br>Or, 'general' for wider comments	**Comments**<br>Insert each comment in a new row.<br>Do not paste other tables into this table, your comments could get lost. Please type directly into this table.
Example 1	16	The question/ answer is not correct – it should be "XXXXXXXXXX" – please include references
Example 2	NEW	The following topic XXXXXX could be included in the game – please include source/ reference information
1		
2		
3		
4		
5		
6		
7		
8		
9		
10		
11		
12		
13		
14		
15		
16		

CwPAMS and Focus Games are developing an educational board game to build awareness and inform healthcare professionals about Antimicrobial Stewardship. We would welcome your review of the questions to be used in the game. We are seeking broad comments about the content and accuracy of the answers within their context.

We are **not** asking you to check for spelling or grammar errors nor are we asking you to comment on the wording or layout of individual questions.

We are seeking comments that the content is factually correct, and that the topics are appropriate.

We would also welcome any suggestion on topics that could be included in the game.

We would appreciate returns by 13th May, absolute deadline 17th May 2021, email: amr@commonwealthpharmacy.org

17		
18		
19		
20		

Insert extra rows as needed.

CwPAMS and Focus Games are developing an educational board game to build awareness and inform healthcare professionals about Antimicrobial Stewardship. We would welcome your review of the questions to be used in the game. We are seeking broad comments about the content and accuracy of the answers within their context.
We are **not** asking you to check for spelling or grammar errors nor are we asking you to comment on the wording or layout of individual questions.
We are seeking comments that the content is factually correct, and that the topics are appropriate.
We would also welcome any suggestion on topics that could be included in the game.
We would appreciate returns by 13th May, absolute deadline 17th May 2021, email: amr@commonwealthpharmacy.org

Case studies

**Comment number**	**Page number** Or, 'general' for wider comments	**Comments** Insert each comment in a new row. Do not paste other tables into this table, your comments could get lost. Please type directly into this table.
Example 1	16	The scenario/ answer is not correct – it should be "XXXXXXXXXX" – please include references
Example 2	NEW	The following topic XXXXXX could be included in the game – please include source/ reference information
1		
2		
3		
4		
5		
6		
7		
8		
9		
10		
11		
12		
13		
14		
15		
16		
17		
18		
19		
20		

Insert extra rows as needed.
